# Exploring the Remote Ties between Helitron Transposases and Other Rolling-Circle Replication Proteins

**DOI:** 10.3390/ijms19103079

**Published:** 2018-10-09

**Authors:** Pedro Heringer, Gustavo C. S. Kuhn

**Affiliations:** Departamento de Biologia Geral, Instituto de Ciências Biológicas, Universidade Federal de Minas Gerais, Belo Horizonte, MG, CEP 31270-901, Brazil; pedrohlt@ufmg.br

**Keywords:** Helitron, rolling-circle replication, mobile genetic element, viral evolution

## Abstract

Rolling-circle replication (RCR) elements constitute a diverse group that includes viruses, plasmids, and transposons, present in hosts from all domains of life. Eukaryotic RCR transposons, also known as Helitrons, are found in species from all eukaryotic kingdoms, sometimes representing a large portion of their genomes. Despite the impact of Helitrons on their hosts, knowledge about their relationship with other RCR elements is still elusive. Here, we compared the endonuclease domain sequence of Helitron transposases with the corresponding region from RCR proteins found in a wide variety of mobile genetic elements. To do that, we used a stepwise alignment approach followed by phylogenetic and multidimensional scaling analyses. Although it has been suggested that Helitrons might have originated from prokaryotic transposons or eukaryotic viruses, our results indicate that Helitron transposases share more similarities with proteins from prokaryotic viruses and plasmids instead. We also provide evidence for the division of RCR endonucleases into three groups (Y1, Y2, and Yx), covering the whole diversity of this protein family. Together, these results point to prokaryotic elements as the likely closest ancestors of eukaryotic RCR transposons, and further demonstrate the fluidity that characterizes the boundaries separating viruses, plasmids, and transposons.

## 1. Introduction

Rolling-circle replication (RCR) proteins are essential components of many genetic elements found in all three domains of life. These proteins can be classified into three different groups according to their main function: (i) Rep proteins (vegetative replication), (ii) Mob proteins/relaxases (conjugation), and (iii) transposases (transposon mobility) [1,2]. Helitrons are the eukaryotic representatives of RCR transposable elements (TEs), found in species from all eukaryotic kingdoms in highly variable copy numbers [3,4]. Their transposition is thought to occur by a mechanism similar to the one proposed for bacterial RCR TEs, like the IS91 family of elements [4,5,6]. Briefly, the Helitron transposase binds to the 5’-end of the element, using one of its two catalytic tyrosines to create a 5′-phosphotyrosine intermediate and a free 3′-OH at the donor site. The leading strand covalently bound to the transposase is displaced, the lagging strand is synthesized, and the second catalytic tyrosine nicks the 3’-end, promoting the formation of a double-strand circle intermediate. The transposase then cleaves the leading strand from the circular intermediate, but this time the second tyrosine cleaves the host’s genome, forming a free 3’-OH which attacks the first 5’-phosphotyrosine linkage. After the 3’-end of the circular intermediate is also joined to the recipient’s free 5’-end, an integrated single-strand “loop” is formed and probably resolved during the host’s genome replication. In addition, it has been recently shown that Helitron transposition shares mechanistic similarities with the replication process used by some circular viruses [7]. Despite some of the differences in their mode of propagation, the main catalytic reaction used by all RCR elements is essentially the same [1].

**Figure 1:**
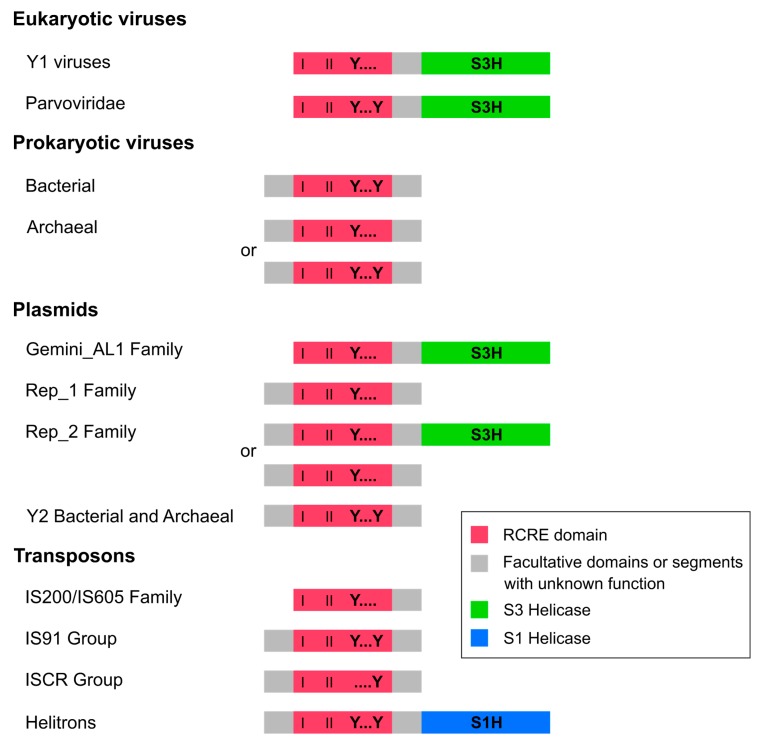
Modular diversity of HUH endonucleases. Schematic representation of the rolling-circle replication (RCR) proteins included in the present analysis. Rolling-circle replication endonuclease (RCRE) domains have the first two motifs (I and II), in addition to the third motif represented by one or two tyrosines (Y) in the catalytic core (dots represent variable amino acid residues). Domains are not drawn to scale, and segments after helicase domains are not represented. Based on information from Chandler et al. [1], Koonin and Dolja [8], and the Conserved Domain Database (CDD) search tool [10].

Helitron transposases are composed of a typical domain, the endonuclease involved in the initiation of RCR (RCRE or Rep), fused to a helicase domain (Hel) from the superfamily 1 (S1H) (Figure 1) [4,8]. This protein, also known as RepHel, belongs to the HUH (named after one of its conserved motifs with two His residues separated by a hydrophobic residue) family of endonucleases [1]. Although HUH endonucleases from eukaryotic viruses and some plasmids also have a helicase domain, they belong to the superfamily 3 (S3H), which is unrelated to the one found in Helitrons. Furthermore, prokaryote viruses only encode a RCRE domain with no helicase (Figure 1) [8,9].

Since Helitrons were discovered [11], a few preliminary suggestions about their evolutive origins have been made. These can be generally divided in two scenarios: the first suggests that Helitrons originated from a prokaryotic ancestral RCR TE [8,11], and the second adds the possibility that Helitrons descended from an ancient eukaryotic viral integration [12]. The first scenario is mainly based on the obvious similarities in the mode of propagation of eukaryotic and prokaryotic RCR TEs, while the second scenario considers the fact that, in contrast to prokaryote RCR TEs, Helitron coding sequences include a helicase domain and sometimes a ssDNA-binding protein, similarly to some RCR proteins from eukaryotic viruses. The fact that many viral copies from geminiviruses were found to be integrated in the tobacco genome [13] was also used to support this hypothesis. In fact, since this scenario was first proposed, several studies showed copies from different eukaryotic RCR viruses in host chromosomes, revealing that viral integrations of these replicons are more common than it was previously thought (reviewed in [14]). In addition, it has been shown that several geminivirus- and parvovirus-related sequences integrated in eukaryote genomes display TE features, and have apparently shifted from a viral to a transposon-like mode of replication [15].

Despite the above considerations, some differences between the RCR proteins of Helitrons and eukaryotic viruses argue against their evolutionary relationship. Firstly, as mentioned before, helicases from these two classes of elements belong to different superfamilies. Also, with the exception of parvoviruses [16], all RCR proteins from eukaryotic ssDNA viruses contain only one tyrosine (Y1) in their catalytic core [9,17], in contrast to the RepHel from Helitrons, which has two (Y2) [4] (Figure 1). Although the number of catalytic tyrosines has been used to tentatively classify RCR proteins between two superfamilies [17], there is currently no phylogenetic support for this distinction. In view of these observations, and considering that domain rearrangements are not uncommon during protein evolution [18], the first scenario (i.e., that Helitrons originated from a prokaryotic ancestral RCR TE) seems to be more parsimonious, as the acquisition of a S1H domain would be the only major evolutionary step in a prokaryotic to eukaryotic RCR TE transition.

The relationship between Helitrons and other RCR genetic elements was initially assessed by Poulter et al. [19]. Although their results did not indicate a relationship between these TEs with specific RCR entities, they provided evidence for an ancient monophyletic origin of Helitrons, which probably occurred early on in the evolution of eukaryotes. However, the evolutionary origin of Helitrons has not been further examined, probably as a consequence of the low sequence identity of RepHel with any other group of RCR proteins [3].

In this study, we investigated the relationship of the Helitron RepHel with other RCR proteins by analyzing the RCRE amino acid sequences from a wide variety of mobile genetic elements, including TEs, plasmids, and viruses. Our results indicate that, despite being eukaryotic TEs, Helitron transposases display more sequence similarities with prokaryotic RCR proteins from bacteriophages and plasmids. In addition, we show that the HUH family of endonucleases can be divided into three major phylogenetic groups comprised of RCR proteins from highly heterogeneous mobile genetic elements. 

## 2. Results and Discussion

### 2.1. Selecting and Preparing RCRE Domain Sequences 

We selected a sample of 13 Helitron RepHel amino acid sequences, representing elements from distantly-related organisms across several phyla and including the main Helitron variants (Table S1). To analyze these TEs in a broad evolutionary context, at least three sequences of each family or group of RCR genetic elements from prokaryotes and eukaryotes were selected. These included single- and double-stranded viruses, plasmids, and TEs (Table S1).

Our analysis was restricted to the RCRE (or HUH) domain of the sequences (Figure 1), which has a central role in starting RCR reactions and is the only region common to all HUH endonucleases [1] (Figure 1). Modular rearrangements often occur during protein evolution [18] which is also the case for several RCR virus lineages [20]. For those reasons, and considering that flanking domains are highly variable amongst RCR elements [1], our restriction to the RCRE domain aimed to avoid spurious evolutionary inferences. Most proteins within the HUH family have three conserved motifs (I, II, and III) in the core region of the RCRE domain, despite the high sequence divergence between groups [1,2,21]. Only amino acid sequences containing all three conserved motifs in their typical arrangement (I-II-III) were selected for our analysis; this is because some HUH endonucleases display their motifs in the reverse order (e.g., III-II-I) [1,2], and these also have highly divergent amino acid sequences, which prevent reliable sequence alignments. A total of 115 amino acid sequences, representing the overall diversity of all known HUH endonucleases, were selected for the analysis (Table S1).

To reduce spurious alignments of the RCRE sequences, we conducted a stepwise alignment approach, which consisted of aligning each group of closely-related sequences separately, excluding segments flanking the RCRE domain and trimming the portions that were exclusive of individual taxa. The resulting sequences (Data S1) were aligned using PSI-Coffee, which is a method considered suitable for highly divergent protein sequences with little or no structural information available [22,23].

### 2.2. Major RCR Protein Phylogenetic Groups

**Figure 2:**
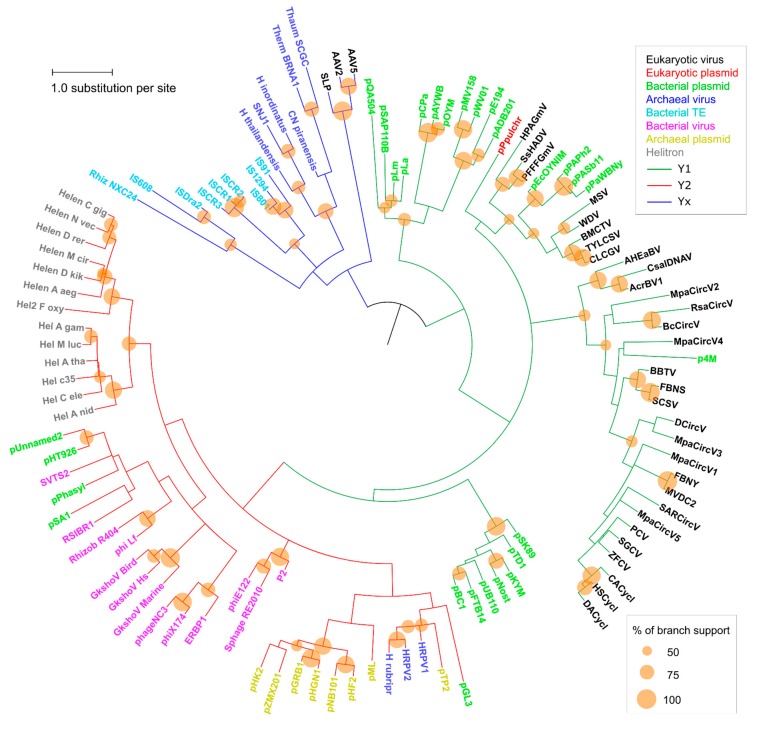
Phylogenetic analysis of RCRE domain sequences. Clade colors indicate each tyrosine group: Y1 (green), Y2 (red), and Yx (blue). Taxa colors represent the family of each element (box on the upper right). See Table S1 for taxa information. Phylogeny inferred by the Maximum Likelihood method (LG+G+I). The same phylogeny, with the numerical support values represented, is shown on Figure S1.

**Figure 3:**
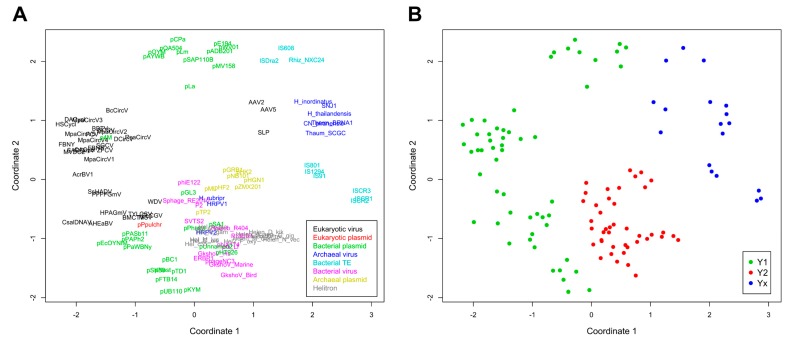
Non-metric multidimensional scaling (NMDS) of evolutionary divergence between RCRE domains. (**A**) Ordinations with taxa represented by their sequence abbreviations. Colors indicate the different classes of mobile genetic elements. (**B**) Same ordinations of (**A**), with colors indicating the tyrosine group of each taxa. The scaling represents euclidean distances for two dimensions (stress: 0.26382).

A phylogenetic analysis was conducted and pairwise divergence values between sequences were used to generate non-metric multidimensional scaling (NMDS) ordinations. As expected for an analysis that includes highly divergent sequences, clade support values between major groups were low, although we observed an overall agreement between our results and the known topology for most of the clades (Figure 2). Our results support the monophyletic nature of all Helitron variants and the lack of any clear relationship of these TEs with other specific groups or families of mobile genetic elements, as previously suggested [19]. Nonetheless, in both the phylogenetic analysis (Figure 2) and NMDS ordinations (Figure 3) we observed an overall distinction between Y1 and Y2 RCR proteins, which we henceforth refer to as Y1 and Y2 groups. An exception is a third clade, composed of elements from both variants, which we refer to here as the Yx group because the number of tyrosines of the catalytic core of its members does not relate with the canonical Y1 and Y2 division. Although the resulting phylogeny revealed a basal segregation of Yx RCR proteins and the rest of the sequences, the Y2 group appears to be more closely related to Y1 RCR proteins, and perhaps constitutes a derivative clade of the Y1 group (Figure 2 and Figure 3).

The topology observed within the Yx group is roughly in agreement with previous results [24], indicating that this clade represents a bona fide phylogenetic cluster composed of archaeal viruses and bacterial TEs. Recent analyses using different methods have also shown that parvoviruses belong to a separate clade from other eukaryotic RCR viruses [25]. However, we did not expect that parvoviral RCR proteins (AAV2, AAV5, and SLP) would group together with Yx elements (Figure 2 and Figure 3). Although structural similarities indicate a distant relationship between parvoviral and other RCR proteins [26], the positioning of these viruses in the Yx group might also be the consequence of long branch attraction [27], so this result should be treated with caution.

As revealed by the results from both analyses, the assignment to a specific catalytic tyrosine group is not contingent on the element class (Figure 2 and Figure 3). For instance, bacterial plasmids, and eukaryotic and archaeal viruses have members in more than one group. Likewise, the element class does not always predict its topology, even within the same tyrosine group. For example, some Y1 viral families are closer to Y1 plasmids than other Y1 viruses, and the same is true in the Y2 group. This phenomenon has been observed in different studies and emphasizes the marked fluidity at the boundaries separating different classes of mobile genetic elements (reviewed in [8,9]). Thus, our results indicate that the tyrosine group division is the only informative phylogenetic feature encompassing the whole HUH endunuclease family.

### 2.3. Helitron Transposase is More Similar to Prokaryotic Proteins

Even though the Helitron RepHel does not appear to be phylogenetically closer to any single family of proteins, they clustered within the Y2 group which, apart from Helitrons, is exclusively composed of prokaryotic viruses and plasmids (Figure 2 and Figure 3). On the other hand, sequences from prokaryotic TEs clustered within the Yx group, even though some of them (including the IS91 family) have two tyrosines in their catalytic core and share a similar transposition mechanism with Helitrons [4,5,6,28]. It is also notable that RepHel proteins appear to be only distantly related to RCR proteins from eukaryotic viruses, which almost exclusively belong to the Y1 group. These observations indicate that the core domain from Helitron transposases is more similar to proteins from prokaryotic viruses and plasmids than to prokaryotic RCR transposases or to eukaryotic viral proteins.

As we have mentioned, in addition to the RCRE domain, RepHel proteins also have a S1 helicase domain (Figure 1); more specifically, this S1 helicase belongs to the Pif1 family [4]. Although Pif1 helicases are present in essentially all eukaryote genomes, they also have been found in some prokaryotes [29,30]. Because all known prokaryotic Y2 RCR proteins lack a helicase, this domain could have been acquired from a prokaryote host by the Helitron ancestor before it colonized the first eukaryote genome. However, considering that Pif1 helicases are ubiquitous in eukaryote genomes and found less frequently in prokaryotes, it seems more plausible that Helitrons acquired their helicase domain from a eukaryotic host. Indeed, a preliminary analysis of Pif1 sequences from Helitrons, eukaryotes, and prokaryotes indicates that the helicase domain from Helitrons is closely related to fungal proteins (Figure S2). Interestingly, the helicase domains from distinct Helitron variants formed separate clusters with different fungal proteins, suggesting that Helitrons acquired their helicase domain from at least two independent events (Figure S2).

**Figure 4:**
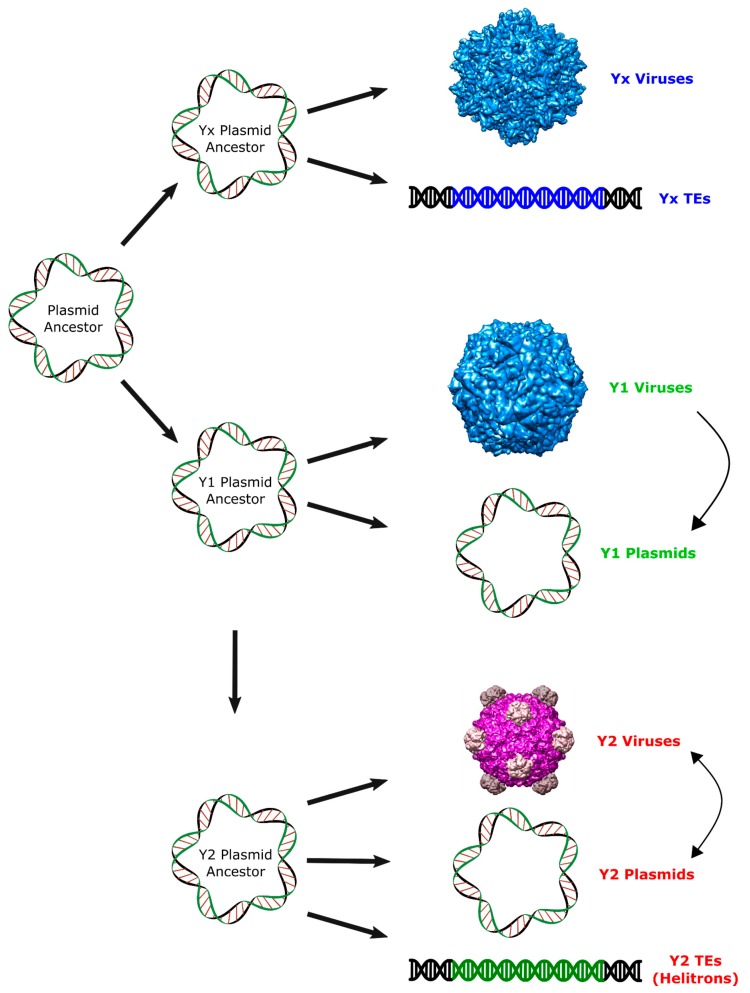
Proposed scenario for the origin of Helitrons and other RCR elements. Arrows represent putative pathways to explain the observed relationship among RCR elements. Virion images were obtained from VIPERdb (http://viperdb.scripps.edu) [31].

These results support the hypothesis of an ancient origin of Helitrons during the initial radiation of eukaryotes, and suggest that neither prokaryotic TEs, nor eukaryotic viruses, are among their closest relatives. Instead, we provide evidence for a closer relationship of these eukaryotic TEs with prokaryotic viruses and plasmids with Y2 RCR proteins, even though it is not possible to determine which specific family shares the most recent common ancestor with the RepHel (Figure 4). Thus, our proposition is that Helitrons descend from a prokaryotic Y2 mobile element that integrated in the genome of an early eukaryote ancestor. Like all other known prokaryotic Y2 elements, the Helitron progenitor probably coded an RCR protein devoid of a helicase domain and was dependent of its host for correct replication/transposition. Subsequently, each of the incipient Helitron variants acquired a eukaryotic helicase by the recombination of its RCRE domain with a host helicase gene. In any case, a comprehensive understanding of the Helitron origins will probably rely on the future discovery of new groups of RCR genetic elements.

Finally, although the RCRE phylogeny does not coincide with the taxonomic division of distinct genetic elements classes (viruses, plasmids and TEs), we suggest that the HUH family of endonucleases is composed by three major radiation groups (Y1, Y2 and Yx). Interestingly, most of the HUH endonucleases can be assigned to one of these groups simply by having a tyrosine residue at a specific position in the RCRE domain, regardless of the element’s class. The extreme diversity observed in each of these groups underscore the dynamic nature of mobile genetic elements which, in the long term, do not evolve under the usual taxonomic constraints acting upon their hosts.

## 3. Materials and Methods

### 3.1. Sequences Retrieval and Selection

RepHel amino acid sequences from Helitrons were retrieved from Repbase (https://www.girinst.org/repbase/) [32] and GenBank (https://www.ncbi.nlm.nih.gov/genbank/) [33], using elements from previous studies as a reference (e.g., [11,19,34]). The structure of these proteins was verified using the Conserved Domain Database (CDD) search tool (https://www.ncbi.nlm.nih.gov/Structure/cdd/wrpsb.cgi) [10]. RepHel sequences that could be clearly assigned to one of the three main Helitron variants [4] were selected: canonical Helitron (6 sequences), Helitron2 (1 sequence), and Helentron (6 sequences). Sequences representing each family or group of RCR proteins were retrieved on GenBank [33], based on several references (e.g., [9,21,24,35,36,37]). A total of 115 amino acid sequences were selected for the alignment (Table S1).

### 3.2. Sequence Alignment

Each family or group of sequences were aligned separately using the M-Coffee mode from T-Coffee (http://tcoffee.crg.cat/) [22] before being manually trimmed in order to exclude flanking portions of the RCRE domain and the segments that are exclusive of individual taxa. The trimmed sequences (Data S1) were aligned with PSI-Coffee (http://tcoffee.crg.cat/apps/tcoffee/do:psicoffee) [22] before manual correction. Alignment positions with less than 90% coverage were excluded.

### 3.3. NMDS and Phylogenetic Analysis

Pairwise evolutionary divergence between sequences was estimated using the Poisson correction model on MEGA7 [38]. The values were used to generate non-metric multidimensional scaling (NMDS) ordinations with the R package vegan [39], representing euclidean distances for two dimensions. NMDS and plotting of ordinations were conducted in RStudio v1.1.442 (Boston, MA, USA) [40]. The best-fit evolutionary model for the alignment (LG+G+I) was determined using MEGA7 [38] and the Smart model selection (SMS) in PhyML (http://www.atgc-montpellier.fr/phyml/) [41]. Maximum Likelihood phylogeny was inferred from 5000 replicates using MEGA7 [38], and the final phylogenetic tree edited using iTOL v4.2.3 (https://itol.embl.de/) [42].

## Supplementary Materials

Supplementary materials can be found at http://www.mdpi.com/1422-0067/19/10/3079/s1.

## Abbreviations

RCR	Rolling-circle replication
TE	Transposable element
RCRE	Rolling-circle replication endonuclease domain
S1H	Superfamily 1 helicase
S3H	Superfamily 3 helicase
RepHel	Helitron transposase (Rep/Helicase)
ssDNA	Single-strand DNA
NMDS	Non-metric multidimensional scaling
