# Supplementary material for: Exploring the Remote Ties between Helitron Transposases and Other Rolling-Circle Replication Proteins
Source: Int J Mol Sci. 2018 Oct 9;19(10):3079. doi: 10.3390/ijms19103079 (PMC6213432; doi:10.3390/ijms19103079)
Supplement: Supplementary file 1 [file ijms-19-03079-s001.pdf]

Supplementary Material

Supplementary Table S1. Taxa information

Group	Sequence ID	Taxon name	Family/Group[a]	# of tyr[b]	Accession
**Eukaryotic viruses**					
	**MSV**	Maize streak virus	Geminiviridae	1	AAF97764.1
	**WDV**	Wheat dwarf virus	Geminiviridae	1	CAA57625.1
	**BMCTV**	Beet mild curly top virus	Geminiviridae	1	AAC54875.1
	**TYLCSV**	Tomato yellow leaf curl Sardinia virus	Geminiviridae	1	CAA43466.1
	**CLCGV**	Cotton leaf curl Gezira virus	Geminiviridae	1	AAF97439.1
	**SsHADV**	Sclerotinia sclerotiorum hypovirulence associated DNA virus 1	Genomoviridae	1	YP_003104796.1
	**PFFFGmV**	Pacific flying fox faeces associated gemycircularvirus 12	Genomoviridae	1	AMH87729.1
	**HPAGmV**	Human plasma-associated gemycircularvirus	Genomoviridae	1	YP_009181996.1
	**BBTV**	Banana bunchy top virus	Nanoviridae	1	NP_604483.1
	**FBNS**	Faba bean necrotic stunt virus	Nanoviridae	1	YP_003104737.1
	**SCSV**	Subterranean clover stunt virus	Nanoviridae	1	Q9ICP7.1
	**FBNY**	Faba bean necrotic yellows C11 alphasatellite	Nanovirus-associated alphasatellite	1	NP_619565.1
	**MVDC2**	Milk vetch dwarf C2 alphasatellite	Nanovirus-associated alphasatellite	1	NP_619760.1
	**PCV**	Porcine circovirus 1	Circoviridae	1	NP_065678.1
	**SGCV**	Silurus glanis circovirus	Circoviridae	1	YP_009091696.1
	**ZFCV**	Zebra finch circovirus	Circoviridae	1	YP_009134739.1
	**HSCycl**	Cyclovirus PK5510 (*H. sapiens*)	Circoviridae	1	ADD62457.1
	**DACycl**	Dragonfly associated cyclovirus 1	Circoviridae	1	YP_009021893.1
	**CACycl**	Chicken associated cyclovirus 1 (NGchicken8)	Circoviridae	1	ADU77011.1
	**DCircV**	Diporeia sp. associated circular vírus	Unclassified[c]	1	AGG39813.1
	**SARCircV**	Circovirus-like genome SAR-A	Unclassified[c]	1	ACQ78172.2
	**MpaCircV1**	McMurdo Ice Shelf pond-associated circular DNA virus 1	Unclassified[c]	1	AIF71501.1
	**MpaCircV2**	McMurdo Ice Shelf pond-associated circular DNA virus 2	Unclassified[c]	1	AIF71504.1
	**MpaCircV3**	McMurdo Ice Shelf pond-associated circular DNA virus 3	Unclassified[c]	1	AIF71507.1
	**MpaCircV4**	McMurdo Ice Shelf pond-associated circular DNA virus 4	Unclassified[c]	1	AIF71509.1
	**MpaCircV5**	McMurdo Ice Shelf pond-associated circular DNA virus 5	Unclassified[c]	1	AIF71512.1
	**RsaCircV**	Rodent stool-associated circular genome virus	Unclassified[c]	1	AEM05803.1
	**BcCircV**	Bat circovirus ZS/China/2011	Unclassified[c]	1	AEL87784.1
	**CsalDNAV**	Chaetoceros salsugineum DNA virus	Bacilladnaviridae[d]	1	YP_473359.1
	**AcrBV1**	Amphibola crenata associated bacilladnavirus 1	Bacilladnaviridae[d]	1	YP_009345107.1
	**AHEaBV**	Avon-Heathcote estuary associated bacilladnavirus	Bacilladnaviridae[d]	1	YP_009345097.1
	**AAV2**	Adeno-associated virus 2	Parvoviridae	2	YP_680422.1
	**AAV5**	Adeno-associated virus 5	Parvoviridae	2	YP_068408.1
	**SLP**	Slow loris parvovirus 1	Parvoviridae	2	YP_009111339.1
**Bacterial viruses**					
	**phiX174**	Enterobacteria phage phiX174	Microviridae	2	NP_040703.1
	**phageNC3**	Enterobacteria phage NC3	Microviridae	2	AAZ49040.1
	**ERBP1**	Eel River basin pequenovirus	Microviridae	2	YP_009126954.1
	**P2**	Escherichia virus P2	Myoviridae	2	NP_046795.1
	**Sphage_RE2010**	Salmonella phage RE-2010	Myoviridae	2	YP_007003504.1
	**phiE122**	Burkholderia virus phiE122	Myoviridae	2	YP_001111165.1
	**phi_Lf**	Xanthomonas phage Lf	Inoviridae	2	AAC54630.1
	**SVTS2**	Spiroplasma phage SVTS2	Inoviridae	2	AAF18311.2
	**Rhizob_R404**	Rhizobacter sp. Root404 (Inovirus Gp2 family protein)	Inoviridae	2	WP_056466193.1
	**RSIBR1**	Ralstonia virus RSIBR1	Inoviridae	2	ATW64834.1
	**GkshoV_Hs**	Gokushovirus WZ-2015a (H.sapiens)	Microviridae	2	ALS03579.1
	**GkshoV_Bird**	Gokushovirus WZ-2015a (Bird)	Microviridae	2	ALS03530.1
	**GkshoV_Marine**	Marine gokushovirus	Microviridae	2	YP_008798246.1

**Archaeal viruses**					
	**HRPV1**	Halorubrum pleomorphic virus 1	Pleolipoviridae	**2**	YP_002791886.1
	**HRPV2**	Halorubrum pleomorphic virus 2	Pleolipoviridae	**2**	YP_005454258.1
	**H_rubripr**	Haloarcula rubripromontorii	Haloarculaceae[e]	**2**	KOX95265.1
	**SNJ1**	Natrinema virus SNJ1	Sphaerolipoviridae	**1**	NC_003158.1[f]
	**H_inordinatus**	Halopelagius inordinatus	Haloferacaceae[e]	**1**	WP_092894117.1
	**H_thailandensis**	Halococcus thailandensis JCM 13552	Halococcaceae[e]	**1**	EMA56448.1
	**CN_piranensis**	Candidatus Nitrosopumilus piranensis	Nitrosopumilaceae[e]	**1**	AJM92193.1
	**Therm_BRNA1**	Thermoplasmatales archaeon BRNA1	unclassified Thermoplasmatales[e]	**1**	WP_015491922.1
	**Thaum_SCGC**	Marine Group I thaumarchaeote SCGC AAA799-P11	unclassified Thaumarchaeota[e]	**1**	WP_048071526.1
**Prokaryotic TEs**					
	**IS91**	Insertion sequence IS91 (*Escherichia coli*)	IS91 Group	**2**	S23782
	**IS801**	Insertion sequence IS801 (*Pseudomonas savastanoi*)	IS91 Group	**2**	P24607.1
	**IS1294**	Insertion sequence IS1294 (*Escherichia coli*)	IS91 Group	**2**	CAA07835.1
	**ISCR1**	Insertion sequence ISCR1 (*Citrobacter freundii*)	ISCR Group	**1**	AFL38296.1
	**ISCR2**	Insertion sequence ISCR2 (*Klebsiella pneumoniae*)	ISCR Group	**1**	SBN37579.1
	**ISCR3**	Insertion sequence ISCR3 (*Pseudomonas aeruginosa*)	ISCR Group	**1**	ATE47644.1
	**IS608**	Insertion sequence IS608 (*Helicobacter pylori*)	IS200/IS605 Family	**1**	2A6M_A
	**Rhiz_NXC24**	IS200/IS605 insertion sequence (*Rhizobium* sp. NXC24)	IS200/IS605 Family	**1**	AVA22184.1
	**ISDra2**	Insertion sequence ISDra2 (*Deinococcus radiodurans*)	IS200/IS605 Family	**1**	WP_010887312.1
**Plasmids**					
**Eukaryotic**	**pPpulchr**	Pyropia pulchra (red algae) plasmid	Gemini_AL1	**1**	AAF36424.1
**Bacterial**	**pEcOYNIM**	Onion yellows phytoplasma EcOYNIM_2000	Gemini_AL1	**1**	YP_006959597.1
	**pPASb11**	Candidatus Phytoplasma australiense plasmid pPASb11	Gemini_AL1	**1**	YP_001965310.1
	**pPAPh2**	Candidatus Phytoplasma australiense plasmid pPAPh2	Gemini_AL1	**1**	YP_001965305.1
	**pPaWBNy**	Paulownia witches'-broom phytoplasma plasmid pPaWBNy-1	Gemini_AL1	**1**	YP_001708784.1
	**p4M**	Bifidobacterium pseudocatenulatum plasmid p4M	Viral_Rep	**1**	NP_613078.1
	**pFTB14**	Bacillus amyloliquefaciens plasmid pFTB14	Rep_1	**1**	P13963.1
	**pUB110**	Staphylococcus aureus plasmid pUB110	Rep_1	**1**	AAA88362.1
	**pBC1**	Bacillus coagulans plasmid pBC1	Rep_1	**1**	AAA98048.1
	**pKYM**	Shigella sonnei plasmid pKYM	Rep_1	**1**	AAA98159.1
	**pSK89**	Staphylococcus aureus plasmid pSK89	Rep_1	**1**	AAB02112.1
	**pNost**	Nostoc sp. plasmid ('pNost')	Rep_1	**1**	AAA25513.1
	**pTD1**	Treponema denticola plasmid pTD1	Rep_1	**1**	AAA98363.1
	**pAYWB**	Aster yellows witches'-broom phytoplasma AYWB plasmid pAYWB-II	Rep_2	**1**	ABC65794.1
	**pOYM**	Onion yellows phytoplasma plasmid pOYM	Rep_2	**1**	YP_002600752.1
	**pCPa**	Candidatus Phytoplasma australiense plasmid pCPa	Rep_2	**1**	YP_001966814.1
	**pLm**	Leuconostoc mesenteroides plasmid replication protein	Rep_2	**1**	WP_002815993.1
	**pLa**	Lactobacillus acidophilus plasmid replication protein	Rep_2	**1**	WP_003549058.1
	**pQA504**	Lactococcus lactis plasmid pQA504	Rep_2	**1**	AEU41945.1
	**pSAP110B**	Staphylococcus epidermidis plasmid SAP110B	Rep_2	**1**	YP_006939186.1
	**pMV158**	Streptococcus agalactiae plasmid pMV158	Rep_2	**1**	YP_001586272.1
	**pE194**	Staphylococcus aureus plasmid pE194	Rep_2	**1**	P03858.2
	**pADB201**	Mycoplasma mycoides pADB201	Rep_2	**1**	NP_040430.2
	**pWV01**	Lactococcus lactis plasmid pWV01	Rep_2	**1**	NP_053450.1
	**pPhasyl**	Phage-plasmid hybrid Phasyl	Phage_GPA	**2**	P19071.1
	**pHT926**	Brevibacillus borstelensis plasmid pHT926	PHA00330	**2**	BAA07788.1
	**pUnnamed2**	Fusobacterium nucleatum subsp. polymorphum plasmid "unnamed2"	PHA00330	**2**	ALQ43495.1
	**pGL3**	Leptolyngbya boryana plasmid pGL3	Unclassified	**2**	AAA25610.1
	**pSA1**	Streptomyces cyaneus plasmid pSA1.1	Unclassified	**2**	BAA34784.1
**Archaeal**	**pHGN1**	Halobacterium sp. plasmid pHGN1	DUF1424	**2**	S06780
	**pGRB1**	Halobacterium salinarum plasmid pGRB1	DUF1424	**2**	P17565.1
	**pZMX201**	Natrinema sp. CX2021 plasmid pZMX201	DUF1424	**2**	YP_232880.1
	**pHF2**	Haloferax sp. Q22 plasmid pHF2	DUF1424	**2**	AKN10606.1

	**pHK2**	Haloferax lucentense DSM 14919 plasmid pHK2	DUF1424	**2**	YP_006961960.1
	**pNB101**	Natronobacterium sp. AS-7091 plasmid pNB101	DUF1424	**2**	NP_942603.1
	**pML**	Methanohalophilus mahii plasmid pML	DUF1424	**2**	NP_976268.1
	**pTP2**	Thermococcus prieurii plasmid pTP2	PHA00330	**2**	YP_007974244.1
**Helitrons**					
	**Helen_A_aeg**	Helitron-2_Aae (*Aedes aegypti*)	Helentron	**2**	Helitron-2_Aae [g]
	**Helen_D_rer**	Helitron-2_DR (*Danio rerio*)	Helentron	**2**	Helitron-2_DR [g]
	**Helen_D_kik**	Helitron-1_DK (*Drosophila kikkawai*)	Helentron	**2**	Helitron-1_DK [g]
	**Helen_N_vec**	Helitron-1_NV (*Nematostella vectensis*)	Helentron	**2**	Helitron-1_NV [g]
	**Helen_M_cir**	Helitron-like sequence (*Mucor circinelloides)*	Helentron	**2**	EPB86818.1
	**Helen_C_gig**	Helitron-10_Cgi (*Crassostrea gigas*)	Helentron	**2**	Helitron-10_CGi [g]
	**Hel2_F_oxy**	FoHeli1 (*Fusarium oxysporum*)	Helitron2	**2**	FoHeli1 [g]
	**Hel_A_tha**	HELITRON1 (*Arabidopsis thaliana*)	Helitron	**2**	AAD15468.1
	**Hel_c35**	Hel_c35 (*Cotesia vestalis bracovirus*)	Helitron	**2**	AEE09607.1
	**Hel_M_luc**	HELIBAT1 (*Myotis lucifugus*)	Helitron	**2**	HELIBAT1 [g]
	**Hel_A_nid**	Helitron-1_AN (*Aspergillus nidulans*)	Helitron	**2**	XP_662882.1
	**Hel_C_ele**	HELITRON1_CE (*Caenorhabditis elegans*)	Helitron	**2**	NP_493834.1
	**Hel_A_gam**	HELITRON1_AG (*Anopheles gambiae*)	Helitron	**2**	HELITRON1_AG [g]

Notes:

[a] Plasmids were classified by their RCRE protein family. Helitrons were assigned to their structural variant according to Thomas and Pritham (2015). [b] Number of tyrosines in the catalytic core. The colors indicate the tyrosine group (Y1 = green, Y2 = red, Yx = blue), as shown in figures 2 and 3C. [c] Sequences representing unclassified viruses were sampled from Zawar-Reza et al. (2014). [d] Family proposed by Kazlauskas et al. (2017). [e] Viral sequence integrated in the genome of indicated taxon. [f] Translated ORF was obtained from nucleotide sequence, according to Wang et al. (2016). [g] Sequences retrieved from Repbase (Bao et al. 2015).

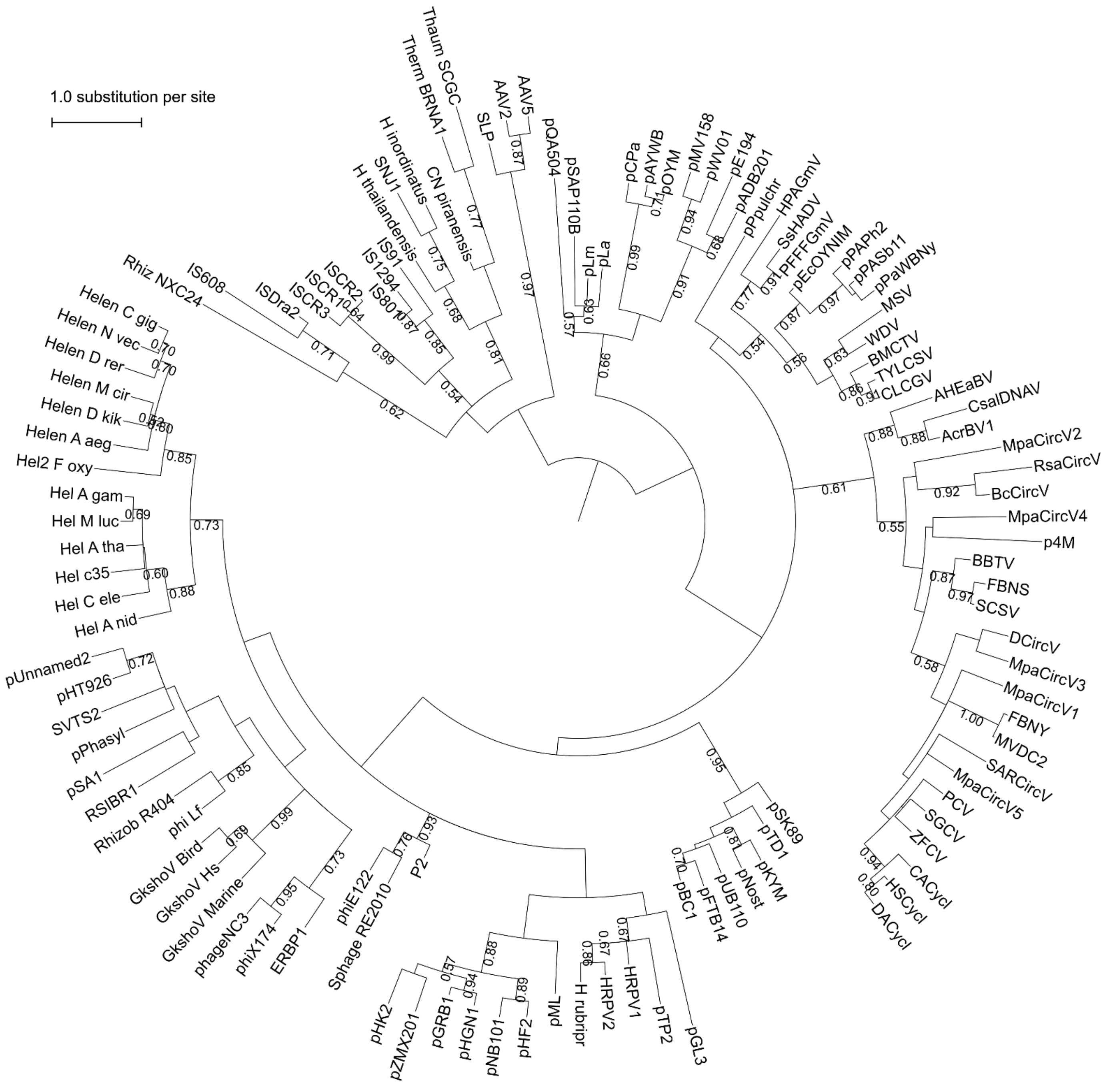

Supplementary Figure S1. Phylogenetic analysis of RCRE domain sequences. Same phylogeny as in Figure 2, with branch support numerical values displayed. Only values above 50% are shown.

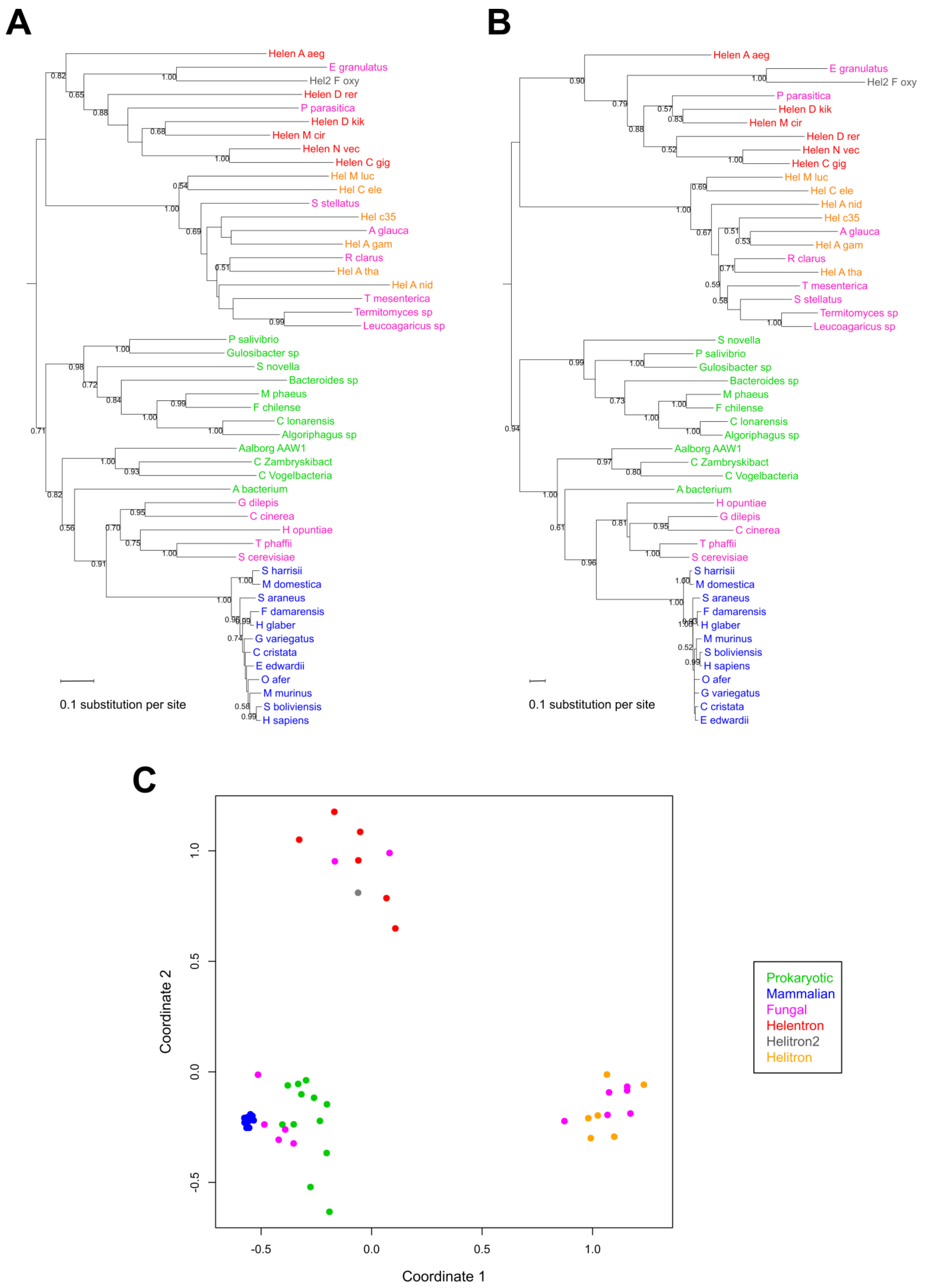

Supplementary Figure S2. Phylogenetic and NMDS analysis of helicase sequences. (A) Phylogeny of helicase domain sequences inferred by the Neighbor Joining method (Poisson correction). (B) Phylogeny of helicase domain sequences inferred by the Maximum Likelihood method (LG+G+I). (C) NMDS of evolutionary divergence between helicase domain sequences with scaling representing euclidean distances for three dimensions (stress: 0.08666). See Table S2 for taxa information.

Supplementary Table S2. Taxa used in the helicase domain analysis [a]

Group	Sequence ID	Taxon name	Accession
Prokaryotes			
	M_phaeus	Myroides phaeus	WP_090404604.1
	F_chilense	Flavobacterium chilense	WP_068841780.1
	C_lonarensis	Cecembia lonarensis	WP_009185623.1
	P_salivibrio	Pontimonas salivibrio	WP_104912779.1
	C_Zambryskibact	Candidatus Zambryskibacteria	OHB14600.1
	Algoriphagus_sp	Algoriphagus sp.	WP_100627322.1
	A_bacterium	Alphaproteobacteria bacterium	OJV13697.1
	C_Vogelbacteria	Candidatus Vogelbacteria	OHA59397.1
	Aalborg_AAW1	SR1 bacterium Aalborg_AAW-1	AKH32407.1
	Gulosibacter_sp	Gulosibacter sp.	WP_087008023.1
	Bacteroides_sp	Bacteroides sp.	CDC65823.1
	S_novella	Starkeya novella	PZQ84937.1
Fungi			
	P_parasitica	Parasitella parasitica	CEP10706.1
	G_dilepis	Gymnopilus dilepis	PPQ64766.1
	C_cinerea	Coprinopsis cinerea	XP_001829007.2
	H_opuntiae	Hanseniaspora opuntiae	OEJ83279.1
	T_phaffii	Tetrapisispora phaffii	XP_003684282.1
	E_granulatus	Elaphomyces granulatus	OXV06635.1
	R_clarus	Rhizophagus clarus	GBB91117.1
	T_mesenterica	Tremella mesenterica	XP_007002293.1
	A_glauca	Absidia glauca	SAL95951.1
	Termitomyces_sp	Termitomyces sp.	KNZ79783.1
	Leucoagaricus_sp	Leucoagaricus sp.	KXN86260.1
	S_stellatus	Sphaerobolus stellatus	KIJ35046.1
	S_cerevisiae	Saccharomyces cerevisiae	NP_013650.1
Mammals			
	F_damarensis	Fukomys damarensis	XP_010639595.1
	H_glaber	Heterocephalus glaber	EHA98492.1
	S_boliviensis	Saimiri boliviensis	XP_010349962.1
	S_araneus	Sorex araneus	XP_004619712.1
	M_murinus	Microcebus murinus	XP_012614176.1
	H_sapiens	Homo sapiens	NP_079325.2
	S_harrisii	Sarcophilus harrisii	XP_012398677.2
	M_domestica	Monodelphis domestica	XP_007479627.1
	G_variegatus	Galeopterus variegatus	XP_008566201.1
	C_cristata	Condylura cristata	XP_004687737.1
	E_edwardii	Elephantulus edwardii	XP_006899697.1
	O_afer	Orycteropus afer	XP_007956003.1
Helentron			
	Helen_A_aeg	Helitron-2_Aae (Aedes aegypti)	Helitron-2_Aae [b]
	Helen_D_rer	Helitron-2_DR (Danio rerio)	Helitron-2_DR [b]
	Helen_D_kik	Helitron-1_DK (Drosophila kikkawai)	Helitron-1_DK [b]
	Helen_N_vec	Helitron-1_NV (Nematostella vectensis)	Helitron-1_NV [b]
	Helen_M_cir	Helitron-like sequence (Mucor circinelloides)	EPB86818.1
	Helen_C_gig	Helitron-10_Cgi (Crassostrea gigas)	Helitron-10_CGi [b]
Helitron2			
	Hel2_F_oxy	FoHeli1 (Fusarium oxysporum)	FoHeli1 [b]
Helitron			
	Hel_A_tha	HELITRON1 (Arabidopsis thaliana)	AAD15468.1
	Hel_c35	Hel_c35 (Cotesia vestalis bracovirus)	AEE09607.1
	Hel_M_luc	HELIBAT1 (Myotis lucifugus)	HELIBAT1 [b]
	Hel_A_nid	Helitron-1_AN (Aspergillus nidulans)	XP_662882.1
	Hel_C_ele	HELITRON1_CE (Caenorhabditis elegans)	NP_493834.1
	Hel_A_gam	HELITRON1_AG (Anopheles gambiae)	HELITRON1_AG [b]

Notes: [a] Prokaryotic, fungal and mammalian sequences were retrieved from Genbank (Benson et al. 2017) by using Helitron sequences as a reference. [b] Sequences retrieved from Repbase (Bao et al. 2015).

References

Bao, W.; Kojima, K.K; Kohany, O. Repbase Update, a database of repetitive elements in eukaryotic genomes. *Mob. DNA* 2015, 6, 11, https://doi.org/10.1186/s13100-015-0041-9.

Benson, D.A.; Cavanaugh, M.; Clark, K.; Karsch-Mizrachi, I.; Lipman, D.J.; Ostell, J.; Sayers, E.W. GenBank. *Nucleic Acids Res.* 2017, 45:D37–D42, https://doi.org/10.1093/nar/gkx1094.

Kazlauskas, D.; Dayaram, A.; Kraberger, S.; Goldstien, S.; Varsani, A.; Krupovic, M. Evolutionary history of ssDNA bacilladnaviruses features horizontal acquisition of the capsid gene from ssRNA nodaviruses. *Virology* 2017, 504, 114-121, https://doi.org/10.1016/j.virol.2017.02.001.

Thomas, J.; Pritham, E.J. Helitrons, the eukaryotic rolling-circle transposable elements. *Microbiol. Spectr.* 2015, 3, MDNA3-0049-2014, https://www.doi.org/10.1128/microbiolspec.MDNA3-0049-2014.

Wang, Y.; Sima, L.; Lv, J.; Huang, S.; Liu, Y.; Wang, J.; Krupovic, M.; Chen, X. Identification, characterization, and application of the replicon region of the halophilic temperate sphaerolipovirus SNJ1. *J. Bacteriol.* 2016, 198:1952–1964, http://dx.doi.org/10.1128/JB.00131-16.

Zawar-Reza, P.; Argüello-Astorga, G.R.; Kraberger, S.; Julian, L.; Stainton, D.; Broady, P.A.; Varsani, A. Diverse small circular single-stranded DNA viruses identified in a freshwater pond on the McMurdo Ice Shelf (Antarctica). *Infect. Genet. Evol.* 2014, 26, 132-138, https://doi.org/10.1016/j.meegid.2014.05.018.

Supplementary Data S1. Trimmed amino acid sequences used in the alignment

>MSV

VNTFLTYPHCPENPEIVCQMIWELVGRWTPKYIICAQEAHKDGDMHLHALLQTEKPVRITDSRFFDIEGFHPNIQSAKSVNKVRDYILKEPL

>WDV

KYLFLTYPQCTLEPQYALDSLRTLLNKYEPLYIAAVRELHEDGSPHLHVLVQNKLRASITNPNALNLRMFHPNIQAAKDCNQVRDYITKEVD

>BMCTV

KNIFLTYPRCSVIKEDALEILKNIPCPSDKLFIRVSQEKHQDGSLHLHALIQFKGKAQFRNPRHFDITHFHPNFQGAKSASDVKQYIEKDGD

>TYLCSV

KNYFLTYPKCDLTKENALSQITNLQTPTNKLFIKICRELHENGEPHLHILIQFEGKYNCTNQRFFDLVSFHPNIQGAKSSSDVKSYIDKDGD

>CLCGV

KNYFLTFPKCSLTKEEALEQIQKISTASNKKYIKICRELHEDGQPHLHVLLQFEGKFKCQNQRLFDLVSFHPNIQGAKSSSDVKSYIDKDGD

>SsHADV

KYVLLTYAQCELDAFRVMDKLSLLGAECIIGREHHEDGGTHLHCFAEFGRKFRSRKADVFDVDGHHPNITSRGTPEKGYDYAIKDGD

>PFFFGmV

RYALLTYAQCDLDPFAVVNHLAELAAECIIGREDHADGGIHLHAFVDFGKKYRTRNTRTFDVEGYHPNISSRRTPEEGYDYAIKDGD

>HPAGmV

RFCIVTYSQTDFDADAIVRILHRDCRGCIVARESHLDGGTHYHAFVDYGTPRDWTNSRRWDVLGVHPNIKVSRTPFNAYAYVGKDKN

>pPpulchr

RLFFLTYPCGLTKELILRELRKIVVVVSKERESGDGYDHFHVLLEAKTKKNYKDPRCFDILGVHGKYETVRNRKRSLKYICKEGD

>pEcOYNIM

QNIFLTYSQCDLSKEEIKTFIINLCNEKKLQINYLIIGIENHQDKGKHHHVFFQLNKQFRTRDLTIFNIPKYSPHIEPIKDTTDVRNYVKKDGD

>pPASb11

KDIFLTYSKCPLGKEKIHNHIKQLMESKNQKIAYIISNTENHQDKEIHTHVLFQLNKRCNLTSQRFFDLDGYHPKIENTRDVEKAIEYIKKDGD

>pPAPh2

RDIFLTYSKCPLGKEKIHNHLKQLLASKKKEIKYIISNNENHQDKEIHTHVFIQLKKQIEITNQRFFDIEGYHPKIETARDVEKSVSYIKKDKD

>pPaWBNy

KDIFLTYSKCPLGKDKIHNHIKQLMASKKKEIQYLITNQENHKDKEIHSHVLFQLTKSATFNGERFFDIEGFHPEIEVARDIEKSISYIKKDGD

>BBTV

VCWMFTINNPTTLPVMRDEIKYMVYQVERGQEGTRHVQGYVEMKRRSSLKQMRGFFPGAHLEKRKGSQEEARSYCMKEDT

>FBNY

KRWCFTLNYKTAVERESFISLFSRDELNYFVCGDETAPTTNQKHLQGYVSLKKMIRLGGLKKKFGYRAHWEIAKGDDFQNRDYCTKETL

>MVDC2

KRWCFTLNYKTALERETFISLFSRDELNYFVCGDEIAPTTGQKHLQGYVSMKKLIRLGGLKKKFGSIAHWEIAKGDDFQNRDYCTKETL

>FBNS

ICWCFTLNNPLSPIFLHESMKYLVYQTEQGESGNIHFQGYIEMKKRTSLAGMKRLIPGAHFEKRRGTQGEARAYAMKEES

>SCSV

ICWCFTLNNPLAPLSLHESMKYLVYQTEAGDNGTIHYQGYVEMKKRTSLVQMKKLLPGAHLEKRRGSQGEARAYAMKEDS

>PCV

KRWVFTLNNPSEEEKNKIRELPISLFDYFVCGEEGLEEGTPHLQGFANFAKKQTFNKVKWYFGARCHIEKAKGTDQQNKEYCSKEGH

>SGCV

KRYVFTLNNYTTEEYARIDNVGADGLARYMITGKEVGENGTPHLQGFINLKVKKRFSQIKEMLGSRCHIEKARGTDLENRVYCSKEGS

>ZFCV

KRWVFTLNNPTEQEVESVKSLPPSEYHYAIVGKEKGEQGTPHLQGFLHLKKKVRLNQMKQLIPRAHFEIARGSDEDNEQYCSKEGD

>HSCycl

RRFCFTWNNYTELNYALCQEFIKKYCKYGIVGKELAPTTNTPHLQGFCNLQKPMRFSTIKKRLDNGIHIEKSMGSDTQNQTYCSKSGE

>DACycl

RRFVFTWNNYTPSDFETCITFLDNFCKYGIIGKEKCPTTQTPHIQGFCNLSKPMRFNNIKKHLHNSIHIEKANGSDEQNKIYCSKSGE

>CACycl

RRFVFTWNNYPIEAYDKCEKYLTKFCKYGIVGEEIAPETGTPHLQGFCNLHKPTRFSTIKKHLDNSIHIEKANGSDIDNQKYCSKSGI

>DCircV

RNWVFTLNNYVDADRVVIGERLANDATYVCYQPEIGASGTPHLQGLVVFANPRTLGGVKRLISDRVHLEPMRGTFAEAHAYCSKDDT

>SARCircV

KAWCFTLNNYTENEHGALVQRFSDFDDKYYFIVGCEIGAQGTPHLQGYIEKKVGRFRPLPCFEVLRDGKNAMHFERAKGNRKQNYNYCSKDGD

>MpaCircV1

KHWQFTLNNPTQDERNVLAELGDQPTTQYLIYGDEVGASGTPHLQGHVSFVQRYRFNQVKNWVSPRAHLELVRLLRRHIEYCKKDGA

>MpaCircV2

RCVCVTIHVDNIFWELQKWNQSLTYGIGQLELGLNGSTHWQMYFENNTAISLTQWKQLLGCKRAHVETRKGTALLAIEYCKKEET

>MpaCircV3

RNFVFTWNNYSDASKTYLSTLACKYVAYAEEVAPTTGTRHLQGFIAFTNAKTIQQARSKLPGCHVETMNGSIAQSEDYCSKAGT

>MpaCircV4

KYWVFTWHGPPKDDEGNRASPALWPEPQFDADMMDALQYQMEIAPSTGKYHYQGAVAFKTRKRSDPLREALAIPGAWTQMMRGSDKDQVYTNKEET

>MpaCircV5

KHWCFTVNNYTDEDIHKLSKASLLLQPLVSSCIYQQEVPGQESATPGTPHLQGFISFKTKQSFKFTKNLVSDRAHVEVAKGTPQQNRIYCSKAKD

>RsaCircV

RYYMLTIPYSLFTIPDPLPEGLVWLKGQPERGENGYEHWQLICCTRKKCRASAVKRLFCPQAHVELTRSAAADEYVWKDDT

>BcCircV

RYWLLTIPYEHFTPYLPPNCAYIKGQLEQGSNTSYLHWQLVVYFSQKKSLNYVKLIFGDGIHCEPSKSKAAEEYVWKEDT

>CsalDNAV

SRCIVTFFPKDNDRRWLKPETYFGPNPDNFQCWCGQFEICPRTGALHAHIYFECVRSRRLRFVRTAALFRKYHHRVHIKKARTVSKKQRQSAINYV
LDDAK

>AcrBV1

GRCIVTLFPPDSEPKWLDPSTYYTDPASVVKIWVGQFEITPETNQIHAHIYIEFHHKKRPKFNLFVKMFTDIGKHVNVKSPKKSNNTQRQGAVNYC
MKDET

>AHEaBV

RSGLLTIHPPSSHPSWLKPETWFPQCDDILEIWCAKFEKGEDTGNLHVHIYFKLKHSNTIRFELLQKWITKHVTGFDFKPQRSATKNSTQCVVNYV
LKPET

>p4M

TDWLLTIRRELPDGSERTVDDVVNALQGIFDAAIGQPEKGEGGYRHYQIFAQGKRQRFSTLKKKLTAAGLGDAHVEPRKGSVSEAVGYCSKEKT

>pAYWB

CELVINANKITKSKIENILELKKKAIQNYAYILHDKDTYQNEKEAQLNGKKIGDLKSPHYHIYLRFNYAYDTKHIAQWFNTQDNFVSKIKGRFSDA
LMYMTHANS

>pOYM

CELVINKTLITKTKIETILETKKKAIQNYAYILHDKDIYQNEKEAQLNGKKVGDIKAPHWHIYLRFNYSQDTKHISQWFNTQENFVSKIKGRFSDA
LMYMIHANR

>pCPa

CELVIKADLIKQTEIEKVLESKKKVIQSYAFILHDNDKYLNEKEAKENGKSVGDYKIPHWHIMLRFHQSQEFKYIAKWFNTTENFVSQIKGRFTDA
LLYLTHANR

>pLm

RTFMYTQQLQHLPFQDVAAFQSRLENINVAEYAFIIHDQDTVDGHPVTSHIHAVLRYQNARSVDSVAKQVSDKAQYIEIWNGNYANAYAYLVHKTD

>pLa

RQFMYTQDLDHLPFKKEDLKTLLEKSSAEEWAYILHDKDIGKNGKTIRPHFHVVMKFKDAKTISRVAKLFNDKQEYIEVWRNTIGNAYSYLIHETS

>pQA504

SVFGFTQQFKADMWDWADDEKAVCFPNGVPDTARIMKRVAERLYVYLIGDIKKANAPDRPHAKDLFKYSAIIHDKDMSFAWDTKTNSKVIVPKELH
MHAVIELPSKRDLSFISTAIGIRPEQIEVPRGRYGRENMLAYLVHAKD

>pSAP110B

TKFMYTQQLKYLNLSIEQLKNNLENDAYIQDFAMINHNKDLDENNQNVAEHLHVFIKLNQQKTIDYVADLVDDKAQYIEFFDKSNKSRNEQNGYLY
LLHKTK

>pMV158

TFLLYPESIPSDWELKLETLGVPMAISPLHDKDKSSIKGQKYKKAHYHVLYIAKNPVTADSVRKKIKLLLGEKSLAMVQVVLNVENMYLYLTHESK

>pE194

TFVLYPESAKAEWLEYLKELHIQFVVSPLHDRDTDTEGRMKKEHYHILVMYEGNKSYEQIKIITEELNATIPQIAGSVKGLVRYMLHMDD

>pADB201

TLLVYPDSAPENWKEILDQNGVEYFGALHDKDVNPDGTIKKPHYHIVLAYSGPTTFNNVKTLCNTLNSPKPLPLDGVGGMWRYMTHKDN

>pWV01

GFLLYPDSIPNDWKEKLESLGVSMAVSPLHDMDEKKDKDTWNSSDVIRNGKHYKKPHYHVIYIARNPVTIESVRNKIKRKLGNSSVAHVEILDYIK
GSYEYLTHESK

>pFTB14

GWIFLTLTVRNVKGERLKPQISEMMEGFRKLFQYKKVKTSVLGFFRALEITKNHEEDTYHPHFHVLLPVKRNYFGKNYIKQAEWTSLWKRAMKLDY
TPIVDIRRVKGRVKIDAEQIESDVREAMMEQKAVLEISKYPVKDTD

>pUB110

RWLFLTLTVKNVYDGEELNKSLSDMAQGFRRMMQYKKINKNLVGFMRATEVTINNKDNSYNQHMHVLVCVEPTYFKNTENYVNQKQWIQFWKKAMK
LDYDPNVKVQMIRPKNKYKSDIQSAIDETAKYPVKDTD

>pBC1

QWLFLTLTVRNTSPESLPETISAMFEGFNRLTKYKAFKTSVKGYFRALEVTKNRDPHSEWFGTYHPHFHVLLCVPSSYFKKKELYITEQEWTDLWK
KAMKLDYTPIVHVQRVKPKEQLEDMETYEEQLKNAIREQNAILEVSKYPVKDTD

>pKYM

RWLFLTLTVRNCEIGELGTVLTAMNAAFKRMEKRKELSPVQGWIRATEVTRGKDGSAHPHFHCLLMVQPSWFKGKNYVKHERWVELWRDCLRVNYE
PNIDIRAVKTKTGEVVANVAEQLQSAVAETLKYSVKPED

>pSK89

QFIFLTLTTPNVTDEHLESEIKNYNHAFQKMFKRKKVNAITKGYVRKLEITYNSKRDDYNPHFHVLMAVNKSYFKDTKAYISQKEWLNLWRDVTGI
SEITQVHVQKIKQNSNKELYEMAKYSGKDSD

>pNost

RWLFVTLTVKNCAITDLRETLTWMNKSFKRFSELKAFPAEGYIKTVEVTRGKTPDGSAHPHFHVLMMVKPSYFGVGYLSQAKWVEMWRKSLRVDYK
PILDVQSLNPQDSLIGLLAEVIKYSVKESD

>pTD1

DFIFITLTVKNCSADELPATLEMMTKGWRRLAMTAMCEFRRSFEGTFKALEITVNKKTGEYHPHYHILAAVKKGYFRKSNPDYISQENLIKLWQKV
CKLDYEPNVDIRRVKNSTYKAVAEVAKYSVKATD

>AAV2

YEIVIKVPSDLDEHLPGISDSFVNWVAEKEWELPPDSDMDLNLIEQAPLTVAEKLQRDFLTEWRRVSKAPEALFFVQFEKGESYFHMHVLVETTGV
KSMVLGRFLSQIREKLIQRIYRGIEPTLPNWFAVTKTRNGAGGGNKVVDECYIPNYLL

>AAV5

YEVIVRVPFDVEEHLPGISDSFVDWVTGQIWELPPESDLNLTLVEQPQLTVADRIRRVFLYEWNKFSKQESKFFVQFEKGSEYFHLHTLVETSGIS
SMVLGRYVSQIRAQLVKVVFQGIEPQINDWVAITKVKKGGANKVVDSGYIPAYLL

>SLP

WELVIKLKYDWIEDLEGSDDPWYDWPEDEIDIYMAILGIKAIKAITRVLRERSKNKTCNYFGQIEQGGEFFHIHLLFEVDGFVSFLLGRMFETLRQ
TLRNSVYFGYPFEVSSEIAITKVKTGGRNKVQDGSYIVNYLL

>SNJ1

HHSVISPPEELYIDAEFPEQELISVAQEFMEEIGMQGIALYHSWSGGDDHDDDIGEWKKRLFADRDWHGDVREELQHRPHVHLIGACPWFPMGDVT
KLTHAETDWVIHRITGKRDGNSSVSLADMRSVARAVVYALSHCA

>H_inordinatus

HHVVFSPPRDWFLQAQDPLDKTFKLIGDILTNHFDAAGRVYYHGWSGGDDLEDDLGEWKNRLFEGRDWETDVRHELEPRPHFHAVVASPFIPGEGV
TDRIHDETGWVIKRIADEKSKRSIDGIDALARVVTYCMSHTS

>H_thailandensis

IHAMFSPEQDWTISRVDGMRSESYELAQEAGVTGGGALLHMWRTTDDLDGEFKKWKYRETYGQGWRQATEVAPHVHQIATAPEFEPEQGDWVAKRV
RTLDAMRSLSHPSSYEDVAGLAMYLLSHTA

>CN_piranensis

LHNIVSIPFELYLTKDGRKKLRAKAIKYLKEFDIDGGVMIDHPYRFSKDLESARLSPHLHLIVTGWLDGQKVKELYEKTGWIVTNVSTIETWNDCY
NLSKYLLSHSA

>Therm_BRNA1

VHVVVSPPQDLRFMRSKEGFRIMVNKVIRVLKDFQVDTGALVFHPWRQCGDRDGSFPSSSFVWRAGPHFHAVGYGYVPEDRIKEFHERTGWILKVV
HDKSDVVSPTATLAYLLTHAG

>Thaum_SCGC

IHLILAVPENQRELPVKLLRQRMSHILKLGNIKGGSVIFHPFRFSKTQHRWYASPHFHLVGFGKSSDIKNAFGRYGWYVKEAGERESVFQTFCYLL
SHCG

>IS91

QHIVFTLPCQYWSLVFHNRWLLAEMSRIAADVILEICHQTDVEPGIFTVIHTWGRDQQWHPHIHLSTTAGGVTSGHTWKNLHFYARKVMSMWRYRI
TRLLSRKYPELVIPDELAVGNSKRDWNCFLDTYRRGWNVNISRVMDNATHVAVYFGSYLK

>IS801

QHLVFTLPDTLWPLFFYNRWLLDALFRLAADNLIYAAKRRGLRVGIFGALHTYGRRLNWHPHVHLSVTAGGLDEQGVWKNLSFHKEALRRRWMWLV
RDYLLGQPLSQLTMPPPLAHILCESDWRRLILAGGQHWHIHLSKKTKNGRKTVNYLGRYLK

>IS1294

VHLVFTLPDTLWPVFESNRWLLNDVCRLAVENLLYAARKRGLEPGIFCAIHTYGRRLNWHPHVHVSVTCGGLNKHGQWKKLSFLKDAMRSRWMWNM
RQLLLKAWSEGMAMPESLSHITTESQWRSLVLKGGKYWHVYMSKKTAGGRNTARYLGRYLK

>ISCR1

RQWVLSFPFQLRFLLARHPQLLSIVYRTLSTHLIKKAGYTKASAQTGSVTLIQRFGSALNLNVHYHMLFLDGVYAEDDYGKQRFHRKALAHTLSHR
IARCMEKRTLTQLHGASVTYRIAVGPQQGRKVFTAGFSLHAGVMAEAHQRDKLERLCRYIS

>ISCR2

RQWVLSFPFQLRFLFASRPEILGIVYRVIATHLVKKAGHTHQVAKTGAVTLIQRFGSALNLNVHFHMLFLDGVYVEQSHGSARFRWKALTHTIAHR
VGRYLERQPMTPLLGHSITYRIAVGSQAGRKVFTAGFSLHAGVAARADERKKLERLCRYIS

>ISCR3

RQWVLSFPYPLRFLFASKPEALGIVQRVIAGWLADQAGIDRASAQCGAVTLIQRFGSALNLNIHFHMLWLDGVYVEATRRELRLHRRALAATIAHR
VCRHLTRKSMDGLRMSSITYRIATGRDAGCKVVTGGFSLHAGVAAEAHESHKLEKLCRYIT

>IS608

HNVVYSCKYHIVWCPKYRRKVLVGAVEMRLKEIIQEVAKELRVEIIEMQTDKDHIHILADIDPSFGVMKFIKRILRQEFNHLKTKLPTLWTNSCFI
STVGGAPLNVVKQYIENQQN

>Rhiz_NXC24

RIVVPDIPHHVTQRGNGRAQTFFCDDDYALYRDLLAHHCRAADVEVWGWVLMPNHVHLILVPADADGIRRALRVHRAYAGHIHARLRRTGHFWQGR
FGCVPMDEEHLAAALRYVALNPV

>ISDra2

RGYVYQLEYHLIWCVKYRHQVLVGEVADGLKDILRDIAAQNGLEVITMEVMPDHVHLLLSATPQQAIPDFVKRRMFVAYPQLKEKLWGGNLWNPSY
CILTVSENTRAQIQKYIESQHD

>phiX174

FIVFDTLTLADDRLEAFYDNPNALRDYFRDIGRMVLAAEGRKANDSHADCYQYFCVPEYGTANGRLHFHAVHFMRTLPTGSVDPNFGRRVRNRRQL
NSLQNTWPYGYSMPIAVRYTQDAFSRSGWLWPVDAKGEPLKATSYMAVGFYVAKYVN

>phageNC3

FFVFDTLTLADDRLQAFNENPNALRDYFRTVGRAVLRAEGRSVKDSYNDCYRYLCVPEFGGQHGRLHWHVVHMVRTLPLGSHDPNFGRKVRNYRQI
NSFRGMWPYGFTQPIAVRYQHDAYSRKGWLWPVDKSGKAMQSKPYQAVAWYVTKYVA

>ERBP1

YCIFNTLTVNESSIEKVFEKGSRIFSDYVRSLDRGVGIAIHKNWRQAVTKRKEGNEFHTYFAVVERGTKNGRLHIHVIHMMKELPNGCVDPNAGRA
IPNRREVTYLKRYWKYGYSAPIAVRFNTNDAFGKKYWRFPVKEVAKNRFESLECKDAGSIIGYIGKYMT

>P2

VGMFITLTAPSKYHPTRQVGKGESKTVQLNHGWNDEAFNPKDAQRYLCHIWSLMRTAFKDNDLQVYGLRVVEPHHDGTPHWHMMLFCNPRQRNQII
EIMRRYALKEDGDERGAARNRFQAKHLNQGGAAGYIAKYIS

>Sphage_RE2010

CAVFYTITCPSRFHSTLNNGRPNPTWTNATVRQSSDYLVGMFAAFRKAMHKAGLRWYGVRVAEPHHDGTVHWHLLCFMRKKDRRAITALLRKFAIR
EDREELGNNTGPRFKSELINPRKGTPTSYIAKYIS

>phiE122

RGVMFTLTCPSRFHAVTTTDSWVRPNPRYDDVDPRAAQAYLRKVWQRTRAELKREGIVYFGMRVAEPNHDGTPHWHGLVFADKIERFCSVMRKHGL
RDSGDEPGAQRHRVRFEMIDRAKGSAVGYVAKYIS

>phi_Lf

AWYFLTLTYRDGSDSSPRDVSELFKRMRGHFNRLKSGRARWNRESFRYVWVGELTQRFRPHYHVMLWVPQGMFFGKVDQRGWWPHGSSQIEKARNC
VGYLAKYAS

>SVTS2

NLSFLTLTYAVNEKDVKKCKNDLKLFFNNINRWWNNPIRSKNHKGILKYMYTYEYQKRGAVHFHIILNQKIPNSVVQQYWKHGINKNIKVRAGSNE
DVVKYLAKYIV

>Rhizob_R404

RPAMLTLTYREVGQWNPKHISDLLQRIRVWVRRRGHGLRYVWVAELQQRGALHYHLLLWLPRGLTLPKPDKQGWWTHGSTRIEWARKPAGYLAKYA
S

>RSIBR1

VTHMITLTTRECITDLDWFLGLWDAFRRAMARYSQFHYIAVPELQKRGAWHMHVAVSGRVALNLARRVWLKVVGGRGKGYCHIRNPQGAHFGKQWK
LDALASYVAKYIG

>GkshoV_Hs

SNYFVTLTYRPDALPYTKDGKPTLRPKDLTNFFKRLRKHKKGNEKIRYFACGEYGEKKGRPHYHVALFNLKLDDLKPLGPSQGYMLYKSKTLQNIW
GLGFVVIGELTYKSASYISRYVM

>GkshoV_Bird

ENYFVTLTYDNDNVPLSQMHMNTLKKRDFQLFMKRLRKRGNDGIRFFACGEYGSTTMRPHYHAILFNLHLDDLEKLYEKDGMVYYTSQFLQSVWKK
GFVIITSMTWETCAYVARYVC

>GkshoV_Marine

SSSFITLTYDNKHLPPNNSLDYTHWQKFIRSLKKRNNGKSIRYFGVGEYGENFGRPHFHAILFGHTFNDLIPMHSNISKSQQLLSAWPRGFVSVGD
VTPESISYVCGYVQ

>HRPV1

SGVMVTLTTDPKRYDSMLDGLMDAWQNLHETLNYLEGTRLDFIRALEFGGSGLPHLHVCVFGVPYIDHRWLKHYWSHAEIVHIHGMNKRGNDSWIM
TSGTHAGKSVAGYLGKYLS

>HRPV2

NAVFCTLTTDPKKFDSLYDAVMSINENFHRLMSYLRSVTGRPRETLDYIKVLEFTSAGYPHLHVLFFDVPWLVDKRELSAKWKQGQIVDLYPLVHR
DDDDWVEEQTRSDDVYQSKTAGSYVGKYIS

>H_rubripr

NAVLVTLTTDPKRQDSLLDGIDSINENLNRLLSYFDSVTGRPRDRPDYIKALEFTEKGYPHLHVLFFDVPWLCDKSEVAAKWAQGEIVDVYPLTYR
DDEDWVRERTRDDGHEKESTAGAYLGKYLS

>pPhasyl

NVGFLTLTFRDHVTDPKEAQRRFNSLKTNILAKRYRAYIRVMEPMKSGRIHYHLLVALHSDIRTGFDFPAVYRQDYSSANKAIRSEWSFWRKTAPK
YGFGRTELMPVRSNSEGIGRYVGKYIS

>pGL3

RLSFITLTLPPAVAEDLSGRWAHVVDLMKRRLPTEIIACTEVQEKVALHLHIVMVGRHSRGSPRQLEKMWSECCETAVRNVIEPNERVTSRVTNSR
TESESNGNGNATGNTSSNANSNGNANGNIHTEVNWNAAVNVQRIKKSASAYMGKYLS

>pHT926

KPVFMTLTFAENVTDVDLANKAFKQFIRKLNGHVYGRGRVGLKYVTVIEFQKRGAVHYHCVFFNLPFIDSGVIASLWGQGFIKVNSMKKRDGTNCD
NVGAYVTKYMQ

>pSA1

PRVFATLTAPELGIPLDPATYDASDLWRYFTIYLRRESRVSFKVAEYQKRGAVHFHAVIRFDGAGDQPARTLHWGTQLDVQPIGAFGHGEEITEQA
VASYVAKYTT

>pUnnamed2

KSTFLTLTFKENIQDIERANREFTLFIKRLKRYLKNQQLKYIATWELQQRGAIHYHLVLFSVPYIDNKKLGELWANGFIKINKIKETVKNEAVGVY
ITKYFV

>pHGN1

HTAMVTLTASTTEEDGGPRPLVDHLRDLLSSWSAVYDALRHTLEDREFEYLAIIEPTPAGYAHIHLGVFVKGPVVAEQFQDVLDAHVKNSEGAGRE
AHRAVVEDDEDEAAVSIRRSARPDREDGIENLGAYLAAYMA

>pGRB1

HTGMVTLTASSTDDEGRLRPPLEHFEDLLESWEAVRRALARVLEGREWEYLAILEPHESGYVHIHLGVFVRGPVVAEQFEPVLDAHLRNCPTAGED
AHQVFDENGDEDAVRVRRSSHPSRSGGVENLGAYLAAYMA

>pZMX201

HTAMLTFTASSRPNGQPIPPVDHLDELLASWDALTTALDRVLGDRRYARLGILEPHNNGYLHIHVAVFIDGKVEQEDFAPVIRSHVNNCEYATEDA
HDPTSEDTISIRHAGDPKRDSDVIGELAIYLAEYLG

>pHF2

TTAMLTLTASHRNEKGGWRCPADHMRDIMDGYDAARKQLHQVLSGRKWEYARVWEPHADGYGHLHIAVFVEDDLRADDFEPVMRSHVENCGPAGSK
AHDPAGDSVSVRDDVENLGSYISEYIG

>pHK2

ATAMLTFTASSVPNGERLPPVEHTDALHDSYDGVRDTLRNTLDADEWGYWLQAEPHNACYSHLHVGVYFDAAVVGPEFERVIDKHVEECEYASFSA
HDYRNTDYLNDSISLNAGVENMGSYLAAYMG

>pNB101

TMVMVTLSASSENAKGGRRCPADHMRDIARGWNSARKALHRVLRRFEWEYAKVWEPHQSGYGHMHVAVAVDDPIEGETFRPVVRSHVENVEPAGSA
AHGLNAVGMGDTVSVNREVENLGSYISEYIG

>pTP2

DAVFLTLTTDPSRFSNLYEANRQFSHSFNRFMSRLRGYFARRGQHLEYIAVYEFTKSGLLHAHVIIFGVRYVISRWWSQGRVVYIYRLRNVDGRWV
WARRRPRDVRAGEGAEDYLKKYLR

>pML

PITMITLTTYQDSQYSVKKHKVDHEQALEMLVDGFRKLRELITRICEGHTPDYFWILEPHESGYPHMHLCYLEEFTEGEQEHIKSIWGAGEQVDFS
FRKPEDTVRSIRNYLMKYMS

>Helen_A_aeg

PTMFLTLSASETQWPLLLKQLHKLTLVNDDAVTCCLYFNKLVDVLMGILSSPRYVVDFFKRIEFQHRGSPHAHIMLWLANDPNETVSELIRKVCSI
SAIHLSETISHTFTCYKRNEKRCRFNIPYWPMNEERTLYEYYLDVLRSSIQRPTIFLKRSMNEMWTNPFNPWIAEKLRSNMDLQFILDVYSCACYL
AGYVN

>Helen_D_rer

PTFFCTFSAAEMRWPEIVTVIKAQEILRSNPVTVMRMFEKRVDALMAHLLLSPEVEDFFYRVEFQARGSPHIHLLAWVKDAPDPEEDNFIDRYVSC
KLPDPNVDPELHKIVTNHSKSCKKGKVVCRFGFPKLPMPKTMITMDDYLNYAEGLTTGSAVLLKRDPKETWVNGYNPDLLRAWNANMDIQYILDAY
SCIMYMLSYVS

>Helen_D_kik

PTFFITFSAAESKWNELLVTLSRLRLIRSDPVTCSRYFDFRFRQLIKLFKSSETLVHYYWRIEFQHRGSPHSHGMYWFSGAPKLEGPEFIDRFITT
TGDDPELQEVIKHSSSCLREGQEFCRFQMPYPPMPETMVLFEEYKFAIRSSLKKPQVFLRRKFSDRLVNAYNRDILGLHRANMDIQFILDAFACCS
YIINYIN

>Helen_N_vec

ATLFCSFSSAETQWMHLLRILGQLRLIQSDPVTCARHFDYQVNQFLTNFLFSSKISDWFYRVEYQQRGSPHIHMLMWLEDAPQFQIDSFIDKIITC
QKPVDNADLLVLVRHSHTCRKNTSSKCRFNYPQPPMKQTMIIKQNYLLAVSSSINTPTVFLKRNPNELRINNYNPDCLSAWRANMDIQFVLDVYAC
AVYIVNYIS

>Helen_M_cir

PTLFITLSAAESKWTELLAMLKKIWLVQSDPVTCASYFDYRFRELKKTRTAPCNVQEYFFRTEFQHRGSPHIHMLIWLEDAPRILPDSFVDGIITC
EKEWDGSPATWDDIIKHTATCKRKDQIVCRFNIPFLPMDVTRVLVDAYIYSIRSTLKTTKVFLRRTPNQVLTNSYNRKILSMFRSNMDLQFIVDGY
ACCSYVADYIN

>Helen_C_gig

PTWFCSFSAAETKWIPLLKTLGKLRLIKSDPVTCSRYFDYRFQRFLHGVLLHKEVVDYFFRVEFQQRGSPHVHMLLWVKNAPNVSSDSFVDRYVSC
SKSGADPVLVRHAKTCMKKNKPICRFNFPIPPMPKTVTLFETYTLAIRSSLTQSKLFLKRQPYEIRINSYNCTLLKSWLANMDIQFILDPYACATY
IVSYIS

>Hel2_F_oxy

PGAFITFSPADLHWRSLYQHMPQYRLLRQNPHIAAFHFYRRYTLFRDIVLSKKSITDYWDRYEWQGRGSPHNHGLYWMDNCPGADMEDTWGFHVTA
INPEPSRTLRLSQIVEAANVANPERECRFDFPRALRELAAVIGRSYYVFEAARNDSLMNNFNPAIILGWLANIDISPCTSLAVITYAAKYCS

>Hel_A_tha

PDLFITFTCNPKWPHITRYCDKRLNPKDRLDIIARIFKIKLDSLMNDLTVKKKTVASMYTVEFQKRGLPHAHILLFMHAKSKLPTSDDIDKLISAE
IPDKEKEPELYEVINVKSPCMVDGECSKLYPKKHQDITKVGSDGYPIYRRRKIDDYVEKGGIKCDNRYVMPYNKKFSLRYNAHINVEWCNQNDSIK
YLFKYIN

>Hel_c35

PDLFITFTCNPKWIEITQLLLPGQTSSDRHDITARIFRQKIRSLMNFIVKQRDTRCWMYSIEWQKRGLPHAHILIWLVERIQPDQIDDIICAEIPD
YEVDPDLHDVVNPQSPCMVDGKCSKRYPRKLTAETVTGNDGYPLYRRRSPDDKVKRMDFVVDNSWIVPYSPLISKSFKTHCNVEYCNSVKSIKYIC
KYVT

>Hel_M_luc

PDLFITMTCNPKWADITNNLQRWQKVENRPDLVARVFNIKLNALLNDICKFHKVIAKIHVIEFQKRGLPHAHILLILDSESKLRSEDDIDRIVKAE
IPDEDQCPRLFQIVNPNSPCMENGKCSKGYPKEFQNATIGNIDGYPKYKRRSGSTMSIGNKVVDNTWIVPYNPYLCLKYNCHINVEVCASIKSVKY
LFKYIY

>Hel_A_nid

PSLFITFTANPAWDEVTRELRPGETWEDRPDIVSRVFNILRAEMVDELCKKKVAPGRFFTIEYQKRGLPHMHLVLFLEERERFLDAAHIDEMVSAE
LPDPREDLELYKLVNSRAPCCDKNMIYCTKRFPKAEQYETQPIEEGYPLYRRRADPRGAYNDMVRIDNTWVVPYNPYLLKRFRSHINVEVCRGVDV
IKYITKYIY

>Hel_C_ele

PDIFLTFTCNPAWTEISENLGPRQSASDRPDLIARVFKLKVVDALFDDLLNRDHVAAYISVFEWQKRGLPHVHMLLTMAENSKPRTSEDIDKIVQA
EIPNPDNEPELHRIVNPHSPCMVDGHCSKRYPKDFHPSTTLNVDGYPGYRRRDDGRYVEYGTQHLDNRRVVPYNKWLLLRYNAHMNVEICGFIEAV
KYLFKYVY

>Hel_A_gam

PDLFITVTCNPKWPEITQCLLPRQQAPDRPDVIVRVFRLKLKAILNDLTMGIEVARIHVIEFQKRGLPHAHILVILAEEDKPQTPADYDKIVSAEL
PNPATSSQLFETVNPAAPCMKDGTCEKGFPKSFCEQTRSMDNGYPQYRRRNNGRSVTVKGIELDNRYVVPYNPWFTHKYNCHINVEVCTSISSVKY
LYKYVY
